# Microbiologically Confirmed Tuberculosis: Factors Associated with Pre-Treatment Loss to Follow-Up, and Time to Treatment Initiation

**DOI:** 10.1371/journal.pone.0168659

**Published:** 2017-01-09

**Authors:** Judith Mwansa-Kambafwile, Boitumelo Maitshotlo, Andrew Black

**Affiliations:** 1 Wits Reproductive Health and HIV Institute, Johannesburg, South Africa; 2 Department of Clinical Medicine, University of Witwatersrand, Johannesburg, South Africa; Fundació Institut d’Investigació en Ciències de la Salut Germans Trias i Pujol, Universitat Autònoma de Barcelona, SPAIN

## Abstract

**Background:**

The impact of new diagnostics on pre-treatment loss to follow up (Pre-treatment LTFU) has not been widely investigated. The reported rate of pre-treatment LTFU is however lower in studies where Xpert MTB/Rif (Xpert) has been used onsite as opposed to centrally. The use of the Xpert at point of care (POC) could have a role in reducing the pre-treatment LTFU rate among TB patients. We aimed to determine the pre-treatment LTFU rate and the time to treatment initiation as well as to describe associated factors in patients diagnosed with TB using POC Xpert or smear microscopy.

**Method:**

Xpert machines were installed at 7 primary healthcare facilities in inner-city Johannesburg. POC Xpert TB testing was the primary diagnostic method for all patients although there were some patients who were tested using only laboratory-based smear microscopy (during power outages or machine operator off-sick). Data on patients’ demographics, TB diagnostic test (Xpert or smear microscopy), test result, and time to treatment initiation were collected. Associations and predictors of pre-treatment LTFU and time to treatment initiation were explored.

**Findings:**

A total of 1981 people with presumptive TB were tested (1743 using Xpert and 238 using smear). A bacteriological diagnosis of TB was made in 271 patients (90% Xpert; 10% smear). The median time to treatment initiation in the smear group was 9 days (IQR: 4–20) while those tested using Xpert had a median time of 0 days (IQR: 0–0). Pre-treatment LTFU was 22.5% with no difference between diagnostic groups (p = 0.8).

**Conclusion:**

The Pre-treatment LTFU rate of 22.5% found in this study is much higher than the 5% target of the South African National TB Control Program. POC Xpert resulted in a significantly greater proportion of bacteriologically proven TB patients being started on treatment within 30 days of presentation. No risk factors associated with pre-treatment LTFU were identified.

## Introduction

Tuberculosis (TB) is a preventable and treatable communicable disease. Despite recent advances in TB diagnostics, TB remains a major contributor to global mortality. With a TB incidence of 834/100000, some of South Africa’s TB patients were among the estimated 1.5 million deaths from the 9.6 million people who developed the disease worldwide in 2014 [1]. Despite the importance of rapid treatment initiation in patients with TB for TB control, neither pre-treatment loss to follow up (LTFU) nor time to treatment initiation are included in routine TB programme outcome measures. TB patients already on treatment have been a focus of TB control programs in many countries. The emphasis has mostly been on ensuring that patients take their treatment. Not much attention has been paid to the patients who test positive for TB but never get initiated on treatment. TB patients who are lost before treatment is initiated continue to transmit infection in communities. This is an important group of people as they contribute to the burden of TB in communities.

Rapid diagnostic methods for TB such as Xpert MTB/RIF (Xpert) have been known to reduce the turn-around time between diagnosis and treatment. Except in a few instances, the testing has been used in centralized laboratory settings [2]. Compared to smear, the use of the Xpert machine in centralized settings has not shown a reduction in mortality or an increase in the notification rate [3–5]. Even when used on site as a “point of care” (POC) test, Xpert has failed to show a reduction in TB mortality and other important TB outcomes [6, 7].

According to the South African National TB Guidelines, pre-treatment LTFU, are laboratory confirmed TB patients who are never commenced on TB treatment [8]. A systematic review which assessed the magnitude of the pre-treatment LTFU rate in smear- or culture-positive TB patients in African and Asian studies found that this rate varies between 4% and 38% [9]. A more recent study conducted in South Africa reported pre-treatment LTFU rates of 14.9 and 17.0% for Xpert and smear microscopy respectively; this was despite telephonic or home visit contact by study investigators at week 1 and month 1 into the trial [5]. Pre-treatment LTFU in a study using Xpert as the initial diagnostic method in a similar setting was 4% [6]. Xpert as a POC may be a means to address high pre-treatment LTFU rates seen in National Tuberculosis Programmes (NTP).

Appropriate TB treatment in a patient rapidly decreases infectivity, decreases transmission and is vital for TB control [10, 11]. Delaying TB treatment initiation or losing bacteriologically confirmed TB patients before treatment is initiated contributes to on-going TB transmission in communities and to poor patient outcomes.

Using smear microscopy, Yimer and colleagues found a 27 day median time delay (between time of patient presentation and time of treatment initiation) that was as a result of health system delays [12]. Although there is a 45% increase in bacteriologically confirmed TB case detection when using Xpert compared to smear microscopy [13] and the turnaround time for TB test results is said to decrease to five days when using laboratory-based Xpert [14], patients are still not initiated on treatment early. Causes of the delays vary from patient factors to health system factors. Factors that have previously been described to increase the risk for these delays are: increased turnaround time between sputum collection and result availability [15], male sex and older age [9]. Cox and colleagues showed that the use of onsite Xpert reduced the time to TB treatment initiation and it decreased pre-treatment LTFU although there was no impact on TB related morbidity or mortality [3].

Implementing POC testing for TB should reduce the impact of the health system factors and therefore reduce the diagnosis-treatment gap [16]. A shortened time to treatment initiation would impact on on-going TB transmission. We aimed to determine the pre-treatment LTFU rate and the time to treatment initiation as well as to describe associated factors in patients diagnosed with TB.

## Materials and Methods

This was a pragmatic observational study within a larger project (Region F TB Blitz) to assess the feasibility of implementing Xpert within primary healthcare facilities (PHCs). Between October 2011 and March 2012, 7 PHCs in inner-city Johannesburg implemented Xpert machines at point of care. These were operated by lay counsellors who had undergone thorough training on operation of the machine. The lay counsellors were funded by the study but were managed by the facility managers as part of the facility staff. In each facility, all persons with presumptive TB (as determined by the TB focal point nurse) were sent to the lay counsellor who collected sputum and tested it for TB using Xpert. Patients were told of the time it would take for a result and were invited to wait at the clinic or return for the result. The management of all patients was at the sole discretion of the TB focal point nurse.

Previous studies reporting on pre-treatment LTFU have used variable cut off times ranging from 30 to 90 days to define pre-treatment LTFU [9]. We were unable to find a fixed cut off time for delayed treatment. However, it is known that there is a dose-response relationship between treatment delay and ongoing transmission of TB. It appears that 30 days is the turning point where a significant increase in the risk for TB transmission occurs [17].

Smear microscopy was the default diagnostic investigation if Xpert was not available at the time the patient presented to the clinic. There were some instances when smear microscopy was the only diagnostic test available at the time. Reasons for this were periods of Xpert machine downtime due to low temperature, electricity outages, software corrupted by viruses and cartridge shortages. The lay counsellors were also not replaced when they were ill or on leave.

As part of the main project and as part of routine data collection in facilities, data on the following were collected: patient demographics, time of TB test, test result, date and time of treatment initiation. The data were captured into an electronic TB register. For this study, pre-treatment LTFU was defined as patients diagnosed with TB and either documented as LTFU before treatment initiation in the TB register or diagnosed with TB but not evaluated (no outcome indicated in the TB identification register and never entered in the TB treatment register). STATA version 12 was used to run descriptive frequencies, proportions, regression analysis and survival analysis. We looked at two cut off times 30 days and 3 months

This research was a sub-study of a larger project called the “Region F TB Blitz” which looked at feasibility of point of care use of Xpert MTB/RIF machine for TB testing. Ethics approval was granted by the University of the Witwatersrand, Human Research Ethics Committee (M110233). Participants of the main study provided written consent for their data to be used for research.

## Results

**Fig 1:**
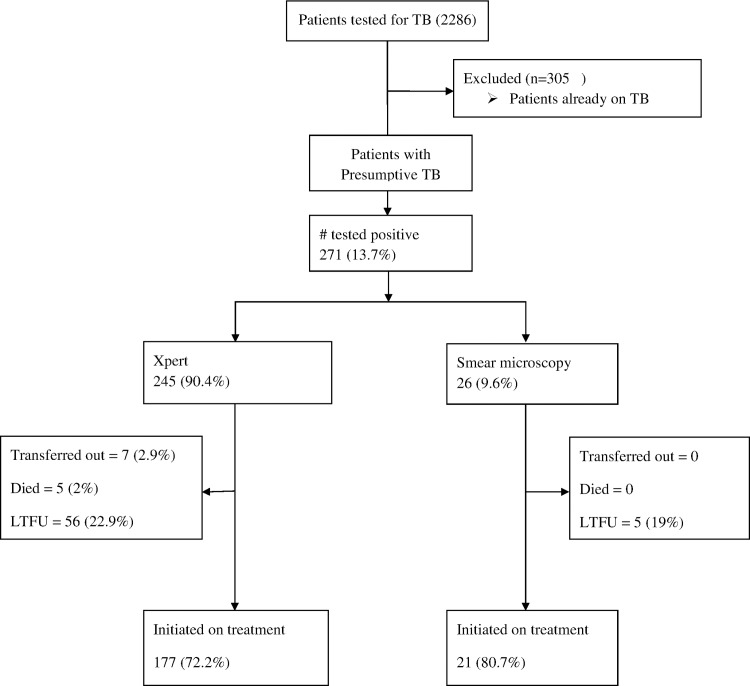
Patient flow and eligibility chart. Patients with presumptive TB tested for TB between October 2011 and March 2012 using either smear microscopy or Xpert were eligible.

A total of 2286 patients were tested for TB during the study period across the 7 PHC facilities. Of these, 13.3% (305) were already on TB treatment and these tests were routine follow up tests as per national TB guidelines for South Africa. The remaining 1981 patients were people who presented with presumptive TB (had one or more of the TB symptoms) (Fig 1).

Although not randomly allocated to diagnostic method (Xpert or smear microscopy) the events resulting in sputum smears being done were random and the participants were similar with regards to age, sex and HIV status Table 1.

**Table 1:** Characteristics of persons suspected of TB by diagnostic test.

	**Tested using Xpert (n = 1743)**	**Tested using smear (n = 238)**	**p-value**
**Age (years; IQR)**	33 (28–39)	34 (29–40)	0.18
**Male n (%)**	880 (51)	135 (57)	0.08
**HIV co-infected n (%)**	1280 (74)	162 (68)	0.06

Two hundred and thirty eight people (12%) had smear microscopy instead of Xpert as the initial diagnostic test.

Overall, the case detection rate was 13.7% (271/1981). The case detection rate for Xpert was 14.1% and for smear microscopy it was 10.9% (p = 0.21).

The proportion of rifampicin resistance among the TB patients tested using Xpert was 6.5%. These patients were referred for MDR TB treatment as per national guidelines.

Of the overall 271 patients that tested positive for TB, 198 (73.1%) were started on treatment; 61 (22.5%) were LTFU prior to starting treatment; 5 (1.8%) died before starting treatment and 7 (2.6%) were transferred out of the facilities. The proportion of patients LTFU pre-treatment among the patients tested using Xpert was similar to the smear microscopy patients (23% versus 19% respectively; p = 0.8).

Fifty two percent (140/271) started treatment within a week of testing positive; and of these 78% (109/140) of them started treatment on the same day. Eighty three percent (146/177) of the patients in the Xpert group were initiated on treatment on the same day of testing while five out of 21 patients (24%) from the smear microscopy group initiated on treatment were started on empiric TB treatment at their initial visit (p = 0.001).

Whether or not treatment was initiated was not significantly associated with age, gender or with the type of test used for diagnosis (Table 2).

**Table 2:** Treatment initiation (within 3 months) in patients with bacteriologically confirmed TB by age, gender and diagnostic method.

		**Adjusted OR**	**95% Confidence interval**
*Age		0.95	0.90–1.03
**Gender**	*Female*	Reference	
	*Male*	0.87	0.50–1.51
**Diagnostic test**	*Smear*	Reference	
	*Xpert*	1.05	0.67–1.67

*Age was analysed as a continuous variable.

**Fig 2:**
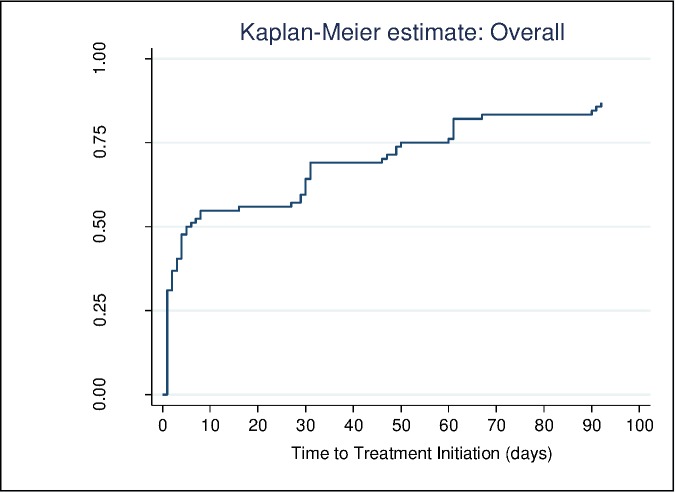
Kaplan-Meier curve showing the time to treatment initiation (in days) for all patients regardless of test method used. It shows the proportion of TB patients initiated on treatment at different time points.

**Fig 3:**
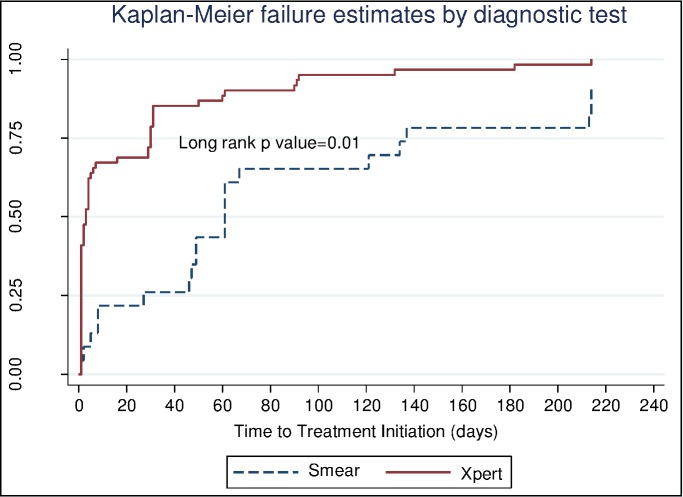
Kaplan-Meier curve showing the time to treatment initiation (in days). The broken line is showing proportion of TB patients diagnosed using smear while the continuous line is for patients diagnosed using Xpert.

About 75% of the patients were initiated on treatment within 2 months of diagnosis and this is regardless of type of diagnostic test used (Fig 2). Patients who tested using the Xpert were initiated on treatment earlier than those tested using smear microscopy (Log-rank p = 0.01) (Fig 3). The median time to treatment initiation for patients that were tested using smear was nine days (IQR: 4–20) while those tested using Xpert had a median time of 0 days (IQR: 0–0). The proportion of patients diagnosed using Xpert and started on treatment before 30 days was different from the proportion from the smear microscopy group started on treatment before 30 days (85% versus 35% p<0.001).

**Fig 4:**
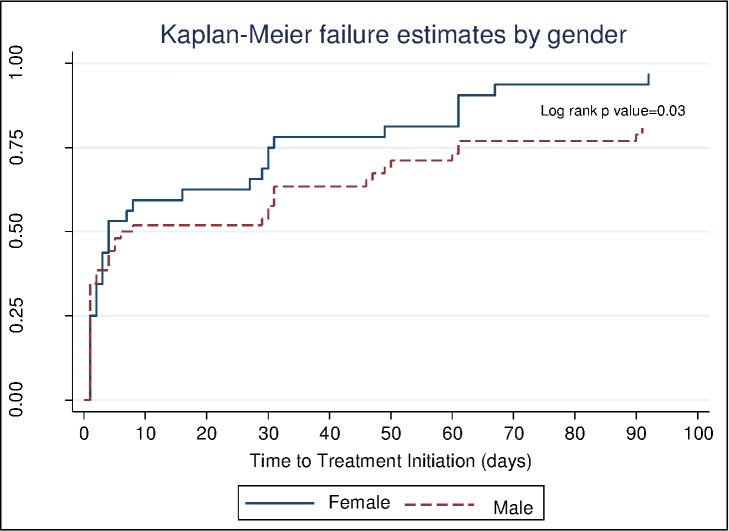
Kaplan-Meier curve showing the time to treatment initiation (in days). It shows proportion of patients initiated on treatment at different time points for males (broken line) and for females (continuous line).

**Fig 5:**
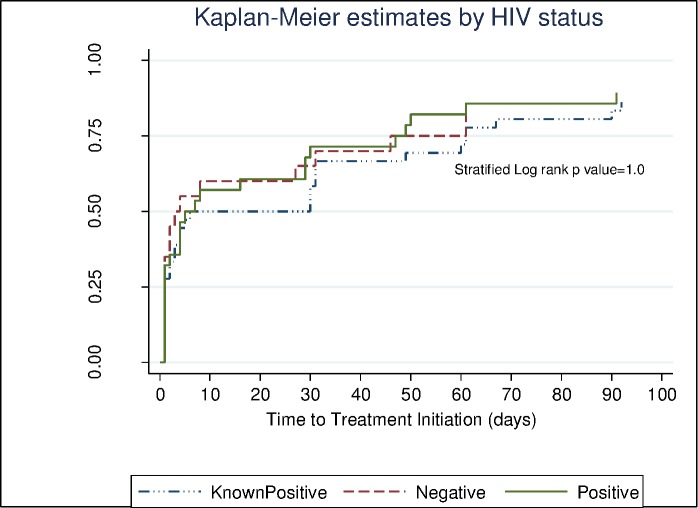
Kaplan-Meier curve showing the time to treatment initiation by HIV status (in days). The 3 groups are HIV positive (continuous line), HIV negative (broken line) and patients with unknown HIV status (broken line with dots).

In terms of duration before treatment initiation, females were initiated earlier than males (Log-rank p = 0.03) (Fig 4). The duration to treatment initiation did not differ among different HIV status categories that is HIV positive, HIV negative and HIV status unknown (Stratified Log rank p = 1.0) (Fig 5).

Twenty seven percent (532/1981) of the patients tested negative for HIV. Thirty six percent of the patients who tested HIV positive were newly diagnosed (516/1449). One of these was labelled as pre-treatment LTFU. Half of the remaining LTFU patients were known HIV positive while the others were HIV negative. However, the pre-treatment LTFU was similar in HIV negative and in HIV positive individuals (p = 0.4).

## Discussion

One of the principles of TB infection control is “prevent formation of infectious TB particles”. Through administrative controls, this can be achieved. Initiating treatment in TB patients rapidly decreases infectiousness. The time to TB treatment initiation and pre-treatment LTFU play a role in the burden of TB in communities. The longer the time before TB treatment is initiated, the greater the risk of transmission of infection to other members of the communities.

Prompt treatment is recognised as an essential part of TB control and has received a lot of focus, but how soon after diagnosis should treatment be started? From a Public Health view delay beyond 30 days is when risk of increased infection becomes significant [17].

In this setting of high HIV and TB prevalence Xpert placed at PHC’s showed a shorter time to treatment initiation when compared to cases diagnosed by laboratory based smear microscopy. These findings are consistent with other studies [3]. Xpert at POC resulted in a significantly larger proportion of the patients who were initiated on treatment at their initial visit. Xpert at POC also resulted in a significantly larger proportion of patients initiated on treatment before 30 days and thus reducing the transmission risk of TB. The difference in time to initiation is unlikely to be as a result of different diagnostic tests and most likely reflects the shorter turnaround time and the readily accessible onsite results that the use of POC Xpert testing enables. The use of onsite technology allows for the gap seen between time of sputum being taken and the result linked to a patient to be overcome. Previous studies have shown that an increase in the turnaround time of diagnostic results increases pre-treatment LTFU [15, 18]. We showed that a greater proportion of patients diagnosed by Xpert were started on treatment within 30 days when compared to laboratory based diagnosis by smear, despite empiric treatment being given to some patients diagnosed by smear microscopy prior to the results being available. The difference in decreased “early” pre-treatment LTFU is important as once treatment is delayed beyond 30 days from diagnosis risk of ongoing transmission increases significantly [17]. As in the TB-NEAT study [3], we found that the impact of earlier treatment initiation on the pre-treatment LTFU rate decreased over time and that proportions of patients initiated on treatment were similar at 54 days in TB-NEAT and 90 days in our study.

The other factor we identified as being associated with delayed treatment initiation was being male. However, this did not translate to a difference in pre-treatment LTFU rate between the 2 gender groups at 90 days.

Despite the availability of onsite results there was no difference in the proportion of patients with bacteriologically confirmed TB started on treatment at 90 days. The high percentage of confirmed TB patients not initiated on treatment in our study (22.5%) is of concern but not unique. An earlier study from Cape Town, South Africa reported 41% of TB suspects with one positive smear microscopy result did not start TB treatment at the PHC facility where they had been diagnosed [19]. We were unable to identify gender, age, or HIV status as risk factors for pre-treatment LTFU.

In areas such as South Africa with high TB/HIV co-infection a high pre-treatment LTFU rate may perhaps be due to early deaths in this patient group as was described by Squire and colleagues [18]. We excluded all known deaths from our analysis but did not check death registries to exclude early deaths in our group of patients with pre-treatment LTFU who could not be traced. We did however not find difference in pre-treatment LTFU between TB/HIV co-infected (either newly diagnosed HIV or known HIV positive) and HIV negative TB patients. Furthermore, the low rate of early deaths seen in the patients who had known outcomes suggests that early deaths are unlikely to explain the high pre-treatment LTFU rate in our study.

We show that it is not only the TB patients who are co-infected with HIV who get lost to follow up. With the strong focus on HIV and TB integration it is important for NTPs to not neglect HIV negative TB patients who may in fact be a larger transmission burden given that they are more likely to have cavitary disease [20]. They also tend to live longer than untreated TB/HIV co-infected patients and so are more likely to transmit infection in the communities.

We have shown a high pre-treatment LTFU rate in our study. Unfortunately this group of patients is excluded from the traditional NTP cohort outcomes analysis and are largely ignored. If TB is to be controlled, steps need to be taken to not only record and report on pre-treatment LTFU but systems need to be put in place urgently to ensure that these patients are traced and appropriately treated.

There are several limitations with this study. It was not designed to compare Xpert TB/Rif with sputum smear. Also, the number of patients in the smear group was small and the diagnostic method used was not randomly allocated to the participants/tests. Lastly, this study was only carried out in Urban clinics and the findings may not represent rural populations. Our results are however consistent with previous findings.

## Conclusion

POC Xpert resulted in a significantly greater proportion of bacteriologically proven TB patients being started on treatment within 30 days of presentation when compared to laboratory based smear microscopy. Pre-treatment LTFU rate was found to be high but was not associated with sex, age or HIV sero-status. NTPs should ensure that they have working systems to manage a patient from time of TB suspicion to treatment completion and attention should be given to decreasing the diagnosis-treatment initiation gap (both time to treatment initiation and pre-treatment LTFU).

## Supporting Information

S1 File Dataset. It shows details of test results of patients.
(XLSX)
